# Acute severe hepatitis outbreak in children: A perfect storm. What do we know, and what questions remain?

**DOI:** 10.3389/fphar.2022.1062408

**Published:** 2022-11-25

**Authors:** Philippa C. Matthews, Cori Campbell, Oana Săndulescu, Mojca Matičič, Simona Maria Ruta, Antonio Rivero-Juárez, Berend Joost van Welzen, Boun Kim Tan, Federico Garcia, George Sebastian Gherlan, Güle Çınar, İmran Hasanoğlu, Ivana Gmizić, Laura Ambra Nicolini, Lurdes Santos, Narina Sargsyants, Petar Velikov, Selma Habibović, Slim Fourati, Snježana Židovec-Lepej, Vanessa Herder, Susanne Dudman, Victor Daniel Miron, William Irving, Gülşen Özkaya Şahin, and ESCMID Study Group for Viral Hepatitis (ESGVH)

**Affiliations:** ^1^ The Francis Crick Institute, London, United Kingdom; ^2^ Division of Infection and Immunity, University College London, London, United Kingdom; ^3^ Department of Infection, University College London Hospitals, London, United Kingdom; ^4^ Nuffield Department of Medicine, University of Oxford, Oxford, England; ^5^ Department of Infectious Diseases, National Institute for Infectious Diseases-Prof. Dr. Matei Balş, Carol Davila University of Medicine and Pharmacy, Bucharest, Romania; ^6^ Faculty of Medicine, Clinic for Infectious Diseases and Febrile Illnesses, University Medical Centre Ljubljana, University of Ljubljana, Ljubljana, Slovenia; ^7^ Virology Department, Stefan S. Nicolau Institute of Virology, “Carol Davila” University of Medicine and Pharmacy, Bucharest, Romania; ^8^ Hospital Universitario Reina Sofía, Instituto Maimónides de Investigación Biomédica de Córdoba, Universidad de Córdoba, Córdoba, Spain; ^9^ Department of Internal Medicine and Infectious Diseases, University Medical Centre Utrecht, Utrecht, Netherlands; ^10^ INSERM U1052, Department of Intensive Care Unit, Hôpital Lyon Sud, Hospices Civils de Lyon, Université Claude Bernard Lyon 1, Lyon, France; ^11^ Microbiology Department, Instituto de Investigacion Ibs.Granada and Ciber de Enfermedades Infecciosas (CIBERINFEC), University Hospital San Cecilio, Granada, Spain; ^12^ Department of Infectious Diseases, “Dr. Victor Babes” Clinical Hospital of Infectious and Tropical Diseases, Bucharest, Romania; ^13^ Department of Infectious Diseases and Clinical Microbiology, Ankara University Faculty of Medicine, Ankara, Turkey; ^14^ Department of Infectious Disease and Clinical Microbiology, Ankara City Hospital, Ankara Yıldırım Beyazıt University, Ankara, Turkey; ^15^ Clinic for Infectious and Tropical Diseases, University Clinical Center of Serbia, Belgrade, Serbia; ^16^ Division of Infectious Diseases , Ospedale Policlinico San Martino, Genova, Italy; ^17^ Nephrology and Infectious Diseases R&D, Infectious Diseases Intensive Care Unit, Faculty of Medicine of University of Porto, Centro Hospitalar Universitário São João, I3S - Instituto de Investigação e Inovaçãoem Saúde, University of Porto, Porto, Portugal; ^18^ Ministry of Health, National Centre for Infectious Diseases, National Institute of Health, Yerevan, Armenia; ^19^ Infectious Diseases Hospital Prof. Ivan Kirov and Department of Infectious Diseases, Parasitology and Tropical Medicine, Medical University of Sofia, Sofia, Bulgaria; ^20^ Department of Microbiology, Public Health Institute Novi Pazar, Novi Pazar, Serbia; ^21^ Department of Virology, INSERM, Henri Mondor Hospital, Assistance Publique-Hôpitaux de Paris, Institut Mondor de Recherche Biomédicale, Université Paris-Est, Créteil, France; ^22^ Department of Immunological and Molecular Diagnostics, University Hospital for Infectious Diseases “Dr Fran Mihaljevic”, Zagreb, Croatia; ^23^ Medical Research Council-University of Glasgow Centre for Virus Research, University of Glasgow, Glasgow, United Kingdom; ^24^ Department of Microbiology, Oslo University Hospital, Institute of Clinical Medicine, University of Oslo, Oslo, Norway; ^25^ National Institute for Mother and Child Health “Alessandrescu-Rusescu”, Carol Davila University of Medicine and Pharmacy, Bucharest, Romania; ^26^ NIHR Biomedical Research Centre, Nottingham University Hospitals NHS Trust and the University of Nottingham, Nottingham, United Kingdom; ^27^ Department of Laboratory Medicine, Section of Clinical Microbiology, Region Skåne, Lund, Sweden; ^28^ Department of Translational Medicine, Lund University, Malmö, Sweden

**Keywords:** paediatric, hepatitis, outbreak, adenovirus, adeno-associated virus, epidemiology, aetiology, liver

## Abstract

During the first half of 2022, the World Health Organization reported an outbreak of acute severe hepatitis of unknown aetiology (AS-Hep-UA) in children, following initial alerts from the United Kingdom (UK) where a cluster of cases was first observed in previously well children aged <6 years. Sporadic cases were then reported across Europe and worldwide, although in most countries incidence did not increase above the expected baseline. There were no consistent epidemiological links between cases, and microbiological investigations ruled out known infectious causes of hepatitis. In this review, we explore the evidence for the role of viral infection, superimposed on a specific host genetic background, as a trigger for liver pathology. This hypothesis is based on a high prevalence of Human Adenovirus (HAdV) 41F in affected children, together with metagenomic evidence of adeno-associated virus (Adeno-associated viruses)-2, which is a putative trigger for an immune-mediated liver injury. Roles for superantigen-mediated pathology have also been explored, with a focus on the potential contribution of SARS-CoV-2 infection. Affected children also had a high frequency of the MHC allele HLA-DRB1*04:01, supporting an immunological predisposition, and may have been vulnerable to viral coinfections due to disruption in normal patterns of exposure and immunity as a result of population lockdowns during the COVID-19 pandemic. We discuss areas of ongoing uncertainty, and highlight the need for ongoing scrutiny to inform clinical and public health interventions for this outbreak and for others that may evolve in future.

## 1 Introduction

**FIGURE 1:**
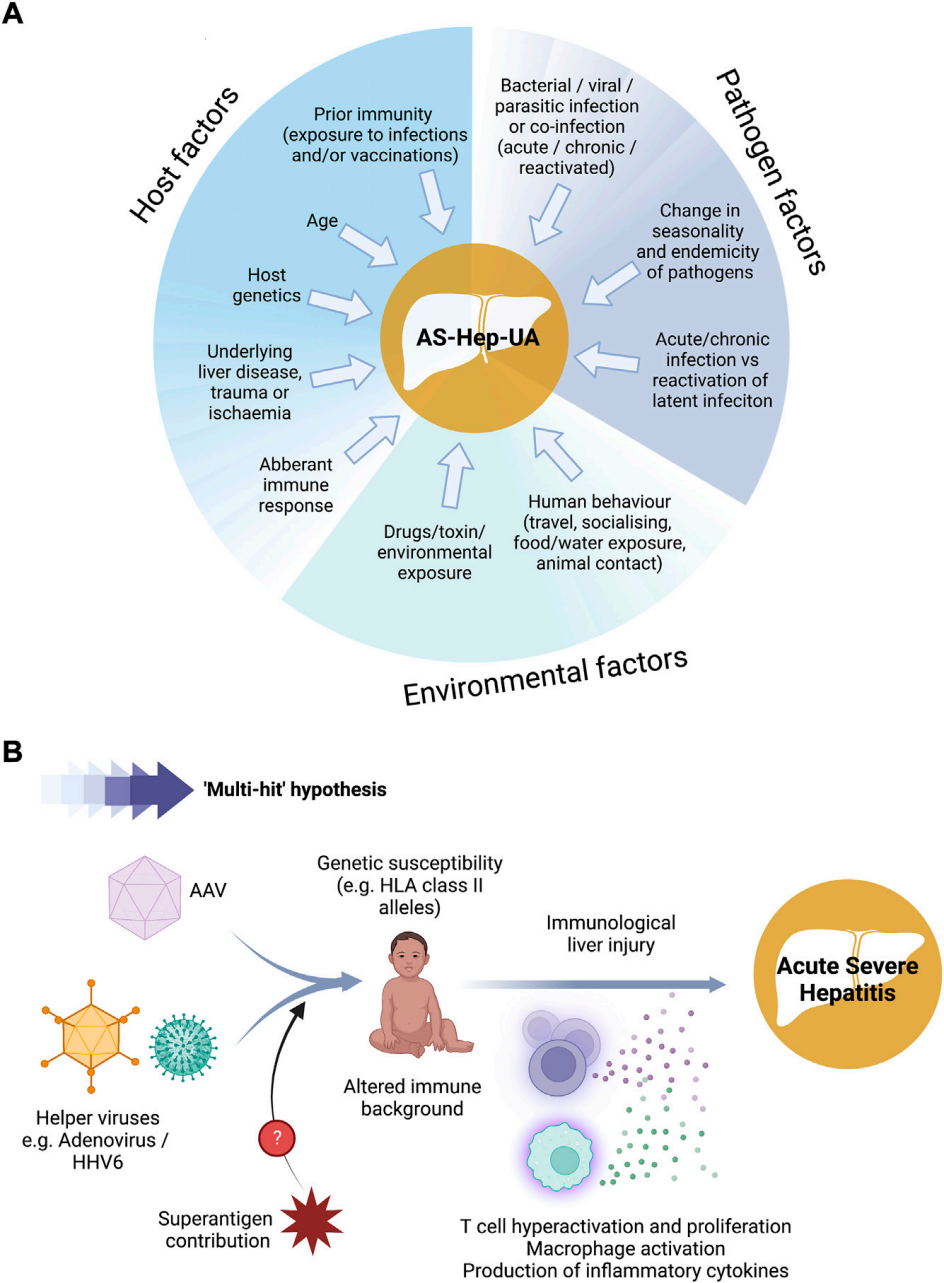
Schematic showing possible factors contributing to the aetiology of AS-Hep-UA in children. **(A)** Possible aetiological agents that have been explored. **(B)** Likely pathogenic pathway underlying paediatric hepatitis outbreak in 2022. AAV - adeno-associated virus; HHV-6 - human herpes virus 6. Figure created using BioRender with a licence to publish.

During the first week of April 2022, the United Kingdom Health Security Agency (UKHSA) alerted the World Health Organization (WHO) to a significant increase in acute severe hepatitis cases occurring in apparently otherwise healthy children under 10 years old, arising since 1st January (WHO, 2022a). A high proportion of these early cases were transferred to paediatric liver units to be evaluated for liver transplant (Marsh et al., 2022). No epidemiologic link between cases was established, no travel outside the United Kingdom was reported, and all tested negative for hepatitis viruses (A to E) and other known causes of acute hepatitis. The aetiology was thus unknown, and the cases were assigned “Acute Severe Hepatitis of Unknown Aetiology” (AS-Hep-UA). Subsequently, additional cases were reported from the European Union/European Economic Area (EU/EEA) countries and globally (ECDC, 2022a), and on 23 April, WHO issued a warning on AS-Hep-UA in children. Since collection of more data is crucial for clarification of the aetiology and pathogenesis of this novel disease, the European Centre for Disease Prevention and Control (ECDC) recommended active searching for cases, and diverse potential causes were investigated (Figure 1A).

As international clinical and epidemiological networks were alerted, multiple countries joined the surveillance effort and more cases started to be reported from around the world, particularly from mainland Europe as well as from the United States. However, the United Kingdom remained the main focus of the outbreak as most cases reported elsewhere were sporadic. Subsequently, the case definition was updated, and more clinical workup data became available from different settings, generating new hypotheses regarding possible aetiologies and pathophysiological processes.

In this article, the European Society for Clinical Microbiology and Infectious Diseases (ESCMID) Study Group for Viral Hepatitis (ESGVH) presents a review of the existing data regarding the epidemiology, aetiology, diagnostics, and ongoing surveillance of AS-Hep-UA. We highlight questions and challenges for ongoing focus as well as lessons to be learned regarding future surveillance efforts and outbreak preparedness.

## 2 Case definition

Initially, the case definitions of AS-Hep-UA differed between countries and time periods (Table 1) (ECDC, 2022b; WHO, 2022b). Criteria included elevated liver enzymes [serum alanine transferase (ALT) or aspartate transferase (AST)], age, and the exclusion of viral hepatitis A-E and other known aetiologies, occurring within a defined time period. The first cases were identified in Scotland, and mostly presented serum aminotransferase levels >2,000 IU/L, reflected in the initial case definition from UKHSA (Marsh et al., 2022). This was subsequently updated as more data became available (Table 1), including cases from Alabama, the United States of America (United States), which were retrospectively noted dating back to October 2021 (Baker et al., 2022). Thus the threshold for aminotransferase levels was modified to >500 IU/L and the date window was extended. By the end of April 2022, WHO and ECDC had started joint surveillance using the European Surveillance System, TESSy (ECDC, 2022c). Although this has unified data collection, there are still some small regional differences in case definition (e.g., the United Kingdom case definition includes only cases <16 years which means cases aged 16 years are not reported) (ECDC, 2022c).

**TABLE 1:** Working case definitions of AS-Hep-UA over time (WHO, 2022a; Baker et al., 2022). All definitions refer to cases of unknown aetiology, thus excluding cases associated with specific infections, drug toxicity, metabolic, hereditary, or autoimmune disorders.

Country and date	Case definition	Description
Scotland, 23 April 2022 (1st technical briefing)	Confirmed	·Serum ALT or AST >500 IU/L without any known cause
		·Age 10 years and under or a contact of any age of a possible or confirmed case
		·Presenting since 1 January 2022
	Possible	·Jaundice without any known cause
		·Age 10 years and under or a contact of any age of a possible or confirmed case, since 1 January 2022
England, Wales, Northern Ireland, 23 April 2022 (1st technical briefing)	Confirmed	·Acute hepatitis (non-hepatitis A-E*) with serum ALT or AST >500 IU/L
		·10 years old and under · Presenting since 1 January 2022
	Possible	·Acute hepatitis (non-hepatitis A-E*)
		·Serum ALT or AST >500 IU/L
		·Age 11–16 years old
		·Presenting since 1 January 2022
	Epi-linked	·Acute hepatitis (non-hepatitis A-E*)
		·Any age
		·close contact of a confirmed case
		·Presenting since 1 January 2022
Scotland, 6 May 2022 (2nd technical briefing)	Confirmed	·Acute hepatitis with a serum ALT or AST >500 IU/L without any known cause (excluding hepatitis A-E, cytomegalovirus and Epstein-Barr virus)
		·10 years of age and under · Or any age if a contact of a confirmed case
		·Presenting since 1 January 2022
England, Wales, Northern Ireland, 6 May 2022 (2nd technical briefing)	Confirmed	·Acute hepatitis with serum ALT or AST >500 IU/L
		·Not due to hepatitis A-E, or an expected presentation of metabolic, inherited or genetic, congenital or mechanical cause**
		·10 years old and under · Presenting since 1 January 2022
	Possible	Acute hepatitis with serum ALT or AST >500 IU/L
		·Not due to hepatitis A-E viruses or an expected presentation of metabolic, inherited or genetic, congenital or mechanical cause**
		·Age 11–15 years
		·Presenting since 1 January 2022
	Epi-linked	·Acute hepatitis (non-hepatitis A-E)
		·Close contact of a confirmed case
		·Presenting since 1 January 2022
WHO and ECDC	Confirmed	N/A at present
	Probable	·Acute hepatitis with ALT or AST >500 IU/L (non-hepatitis A-E*)
		·16 years old or younger
		·Presenting since 1 October 2021
	Epi-linked	·Acute hepatitis (non-hepatitis A-E*)
		·Any age
		·Close contact of a probable case
		·Presenting since 1 October 2021
	Discarded	·A subject previously classified as case, that following further investigations did not meet the case definition criteria

AST: aspartate aminotransferase, ALT: alanine aminotransferase, Epi-linked: epidemiologically linked *If hepatitis A-E serology results are awaited, but other criteria were met, these can be reported and classified as “pending classification”. **Confirmed and possible cases should be reported based on clinical judgment if some hepatitis A-E virus results are awaited, or if there is an acute on chronic hepatic presentation with a metabolic, inherited or genetic, congenital, mechanical, or other underlying cause. If hepatitis A-E serology results are awaited, but other criteria were met, these are classified as “pending classification”.

## 3 Epidemiology

**FIGURE 2:**
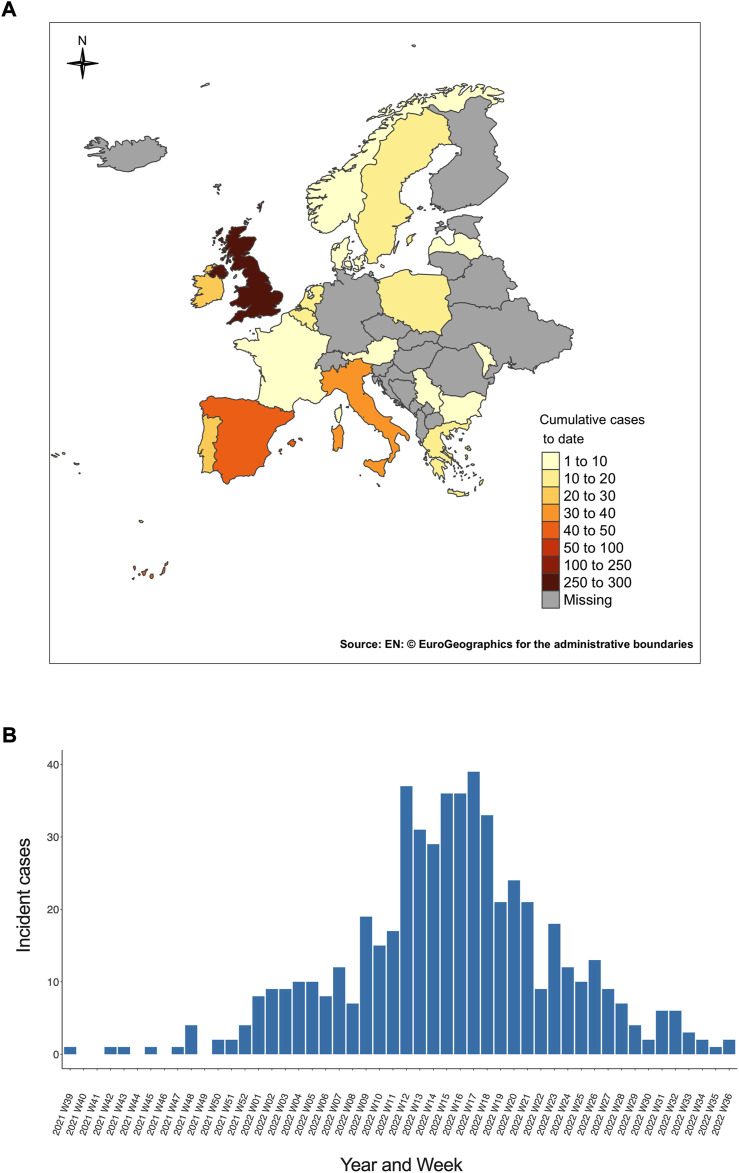
Epidemiology of AS-Hep-UA in Europe. **(A)** Number of cumulative AS-Hep-UA cases reported in European countries by the European Centre for Disease Prevention and Control (ECDC) as of 25 August 2022. Cumulative case numbers are presented for countries for which data was available from the ECDC, and labelled as ‘missing’ for those for which no data were reported. **(B)** Number of weekly probable AS-Hep-UA cases of unknown origin in European children reported between the 39th week of 2021 and the 36th week of 2022. Underlying data for both figure panels sourced from European Centre for Disease Prevention and Control (ECDC) (ECDC, 2022d).

Following initial reports from the United Kingdom, nine children meeting the case definition were retrospectively identified from the United States (Gutierrez Sanchez et al., 2022), and Japan’s health ministry reported a case from Asia. By the end of April 2022, further cases had been reported from Europe, (Zhang et al., 2022). WHO data between 5 April and 8 July 2022 include data for 1010 probable cases from 35 countries representing five WHO regions, predominantly affecting Europe (Figure 2A). These cases are sporadic, with no unifying epidemiological connections, and children have no relevant travel history outside their country of origin. The epidemic peak was reached between week 12 and week 18 of 2022, and subsequently declined steadily (Figure 2B).

However, these data need to be benchmarked carefully against baseline rates of acute unexplained paediatric hepatitis. The initial presentations in Scotland over a 3 week period exceeded the expected annual average for unexplained paediatric hepatitis cases (Marsh et al., 2022). Likewise, elsewhere in the United Kingdom, and in settings within Italy, Spain, Sweden, Ukraine, and Israel, presentations have been higher than expected based on background rates (van Beek et al., 2022), alongside higher rates of liver transplantation (Kelgeri et al., 2022). In contrast, case numbers in India, Japan, Brazil, the United States and elsewhere have not exceeded baseline, and despite the Alabama reports, the United States’ Centres for Disease Control and Prevention (United States CDC) evaluation has found no evidence of an increase in paediatric hepatitis or liver transplantation above levels recorded prior to the COVID-19 pandemic (Kambhampati et al., 2022).

The index cases reported from the central region of Scotland were mostly between 3–5 years-old (65.4%), with a median age of 3.9 years, 54% were female and all were of white Scottish ethnicity (Marsh et al., 2022). Ongoing surveillance has collected data from patients aged from 1 month to 16 years; in the United Kingdom the median age has remained 3 years [interquartile range (IQR) 2–5 years] and 49% are female, 88% are of white ethnicity, and children have been otherwise previously healthy (GOV.UK, 2022).

**FIGURE 3:**
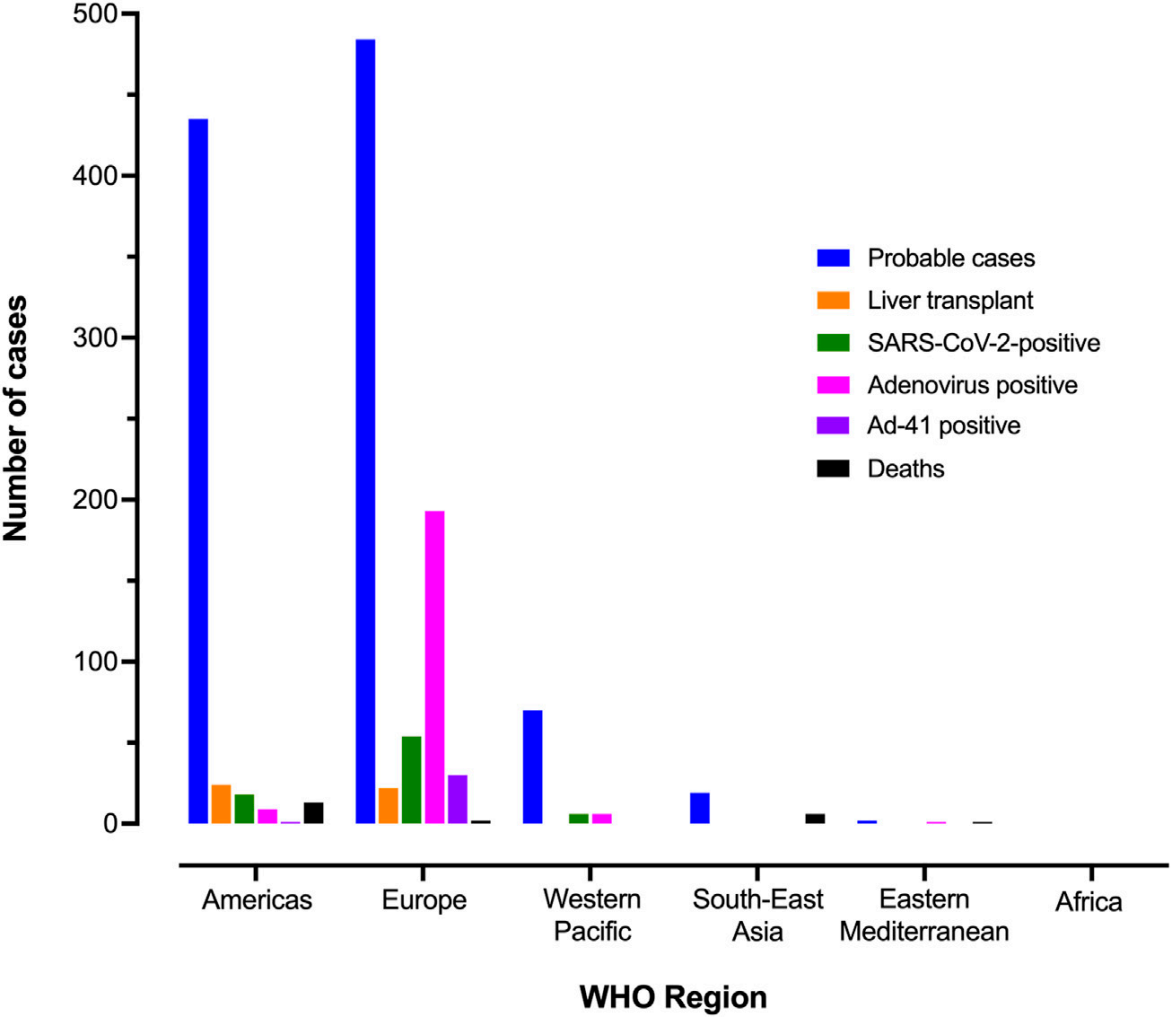
Region of origin of AS-Hep-UA cases and outcomes. Data collected by TESSy for 513 cases of AS-Hep-UA from 21 different countries (Joint ECDC-WHO Regional Office for Europe Hepatitis of Unknown Origin in Children Surveillance Bulletin).

From among 1010 children worldwide meeting the criteria for a “probable” case, 46 (5%) children required liver transplant, and 22 (2%) deaths were reported (Figure 3) (WHO, 2022c). One of the concerning features was the potential for fulminant hepatitis and associated mortality, but it is still unknown if unnoticed mild or clinically asymptomatic cases may also occur, which are not detected by ongoing public health surveillance. To understand the epidemiology more fully, it will be necessary to consider a modified case definition that also captures milder cases (Gutierrez Sanchez et al., 2022; Lurz et al., 2022).

## 4 Screening tests

All patients meeting the case definition should be promptly investigated to increase the potential of identifying an aetiological agent, using a range of specimens in parallel (blood, respiratory samples, stool and urine), ideally with sequential sampling (at presentation, in case of deterioration, pre- and post-intervention, and at recovery).

### 4.1 Routine clinical investigations

First-line investigations include markers of liver cell injury based on elevated liver enzymes. Investigations should first exclude hepatitis A-E viruses using serology: anti-HAV IgM, HBsAg (plus reflex testing for anti-HDV, if HBsAg positive), anti-HBc IgM, anti-HCV antibodies and anti-HEV IgM, and nucleic acid amplification tests (NAAT) for HAV, HCV and HEV. If these are negative, a detailed bank of tests is then recommended for other pathogens and toxins (Table 2), and to exclude autoimmune and metabolic liver disease. In all cases, all specimens should be collected and frozen at <−20°C or if possible at <−80 °C for future investigations (with samples sent to reference laboratories where required to support additional investigations). Children presenting with AS-Hep-US have tested negative for autoimmune hepatitis, metabolic disease, and there has been no evidence to support traumatic, hypoxic or thrombotic liver injury.

**TABLE 2:** Specimen collection and suggested investigative diagnostics (adapted from https://www.who.int/publications/i/item/who-unkhep-laboratory-2022.1). SARS-CoV-2: Severe acute respiratory syndrome coronavirus-2; CMV = cytomegalovirus; EBV = Epstein-Barr virus; HSV-1 and 2 = herpes simplex virus type 1 and 2; VZV = varicella-zoster virus; HHV-6 and 7 = human herpesvirus 6 and 7; RSV = respiratory syncytial virus; NAAT = nucleic acid amplification test.

Specimen type	Test	Pathogens
Whole blood	NAAT	SARS-CoV-2, adenovirus, enterovirus, parechovirus, CMV, EBV, HSV-1, HSV-2, VZV, HHV-6, HHV-7, parvovirus B19, *Leptospira spp*. and metagenomics
	Serology	HIV, rubella virus, anti streptolysin O titre, *Leptospira spp.* and *Coxiella burnetti*
	Culture	Standard culture for bacteria/fungi
	Toxicology	Local investigations according to medical history and geography
Throat swab (oro/naso-pharyngeal)	NAAT	Respiratory virus panel (including adenovirus, human bocavirus, influenza, parainfluenza, rhinovirus/enterovirus, RSV, metapneumovirus, SARS-CoV-2), *Mycoplasma spp.*
	Culture	Standard bacterial panel, including *Streptococcus* group A
Stool	NAAT	Adenovirus, astrovirus, enterovirus, rotavirus, norovirus, sapovirus, enterovirus
	Culture	Standard bacterial stool pathogen panel, including *Salmonella spp.*
Urine	NAAT	Adenovirus, *Leptospira spp.*
	Culture	Standard bacterial urine culture
	Toxicology	Local investigations according to medical history and geography
Liver tissue	Metagenomics	All pathogens

### 4.2 Expanded investigation with metagenomics

Where possible, additional testing is recommended using a metagenomic approach to investigate all possible microbiological aetiologies (focusing on blood and liver biopsy when available). Metagenomics takes a universal (pathogen-agnostic) approach, by sequencing all genetic material found in a specimen, thus potentially retrieving sequences of both known and unknown microbes, including organisms that have not previously been described as pathogenic in humans. Such approaches are not routinely available in clinical diagnostic labs and not validated for clinical diagnostics, so typically require support from research facilities and/or reference laboratories, and any potentially relevant organism identified by metagenomics should ideally be confirmed using standard methods (culture, NAAT and/or genome sequencing).

### 4.3 Histopathological examination

**FIGURE 4:**
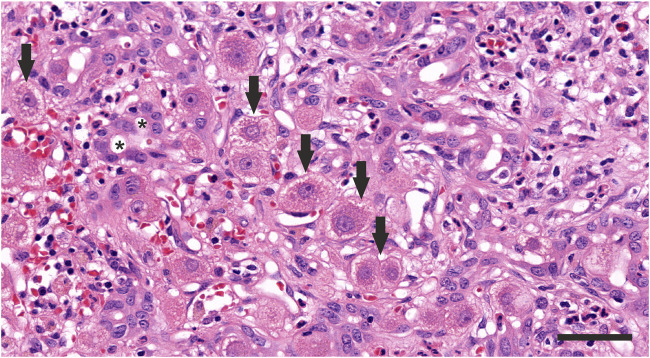
Histopathology of the liver of a child in the United Kingdom with AS-Hep-UA in which AAV-2 was detected. Severe hepatitis is present, with cytoplasmic vacuolation of hepatocytes (arraws) as well as a prominent proliferation of the bile ductules (*) and infiltration with neutrophils, lymphocytes, plasma cells, and macrophafes. HE staining, scale bar 50 μm.

In some cases, liver biopsy or explant has been undertaken, allowing examination for specific causes of liver injury and deriving a tissue phenotype for AS-Hep-UA (Figure 4). Examination has revealed mild diffuse inflammatory infiltrates of lymphocytes (predominantly CD8^+^ T cells, in addition to CD4^+^ T cells and B cells), plasma cells, and eosinophils, alongside characteristic changes of hepatocyte ballooning, canalicular cholestasis, and scattered apoptotic bodies (Ho et al., 2022; Kelgeri et al., 2022; Morfopoulou et al., 2022). Explant tissue was characterised by submassive necrosis with extensive macrophage infiltration, while panacinar necrosis was observed in tissue from recovered children. Importantly, histological examination has not identified any viral inclusions, viral antigens, viral nucleic acid or viral particles of either HAdV or AAV nor any evidence of underlying chronic liver disease (Kelgeri et al., 2022).

## 5 Infective aetiology of AS-Hep-UA

To date, the working hypothesis is that AS-Hep-UA is most likely triggered by an infection or combination of infections (Figure 1B), but ongoing surveillance and epidemiological investigations remain crucial (Sallam et al., 2022). Extensive analysis has not identified any consistent epidemiological links, relevant common exposures, or associations with previous immunosuppression. In this section we review the potential aetiological agents that have been explored, and briefly present the evidence for and against the involvement of these triggers in the AS-Hep-UA outbreak (case series are summarised in Table 3).

**TABLE 3:** Summary of data presented from United States, United Kingdom and European case series of children with AS-Hep-UA.

Cohort and citation	Clinical outcomes	Liver histology	Microbiological investigations
9 children with acute hepatitis in Alabama, United States; Baker et al. (2022); Gutierrez Sanchez et al. (2022)	2/9 transplanted. No deaths	Liver biopsy from 6 patients: various degrees of hepatitis with no viral inclusions, no evidence of adenovirus (immunohistochemistry), no viral particles (electron microscopy)	> Whole blood: adenoviral RNA positive in all patients (adenovirus type 41 in 5 specimens)
			> Other viruses detected: EBV, enterovirus/rhinovirus, metapneumovirus, RSV, human coronavirus OFC43
13 children (median age 3.9 years, M/F: 5/7) in Scotland; Marsh et al. (2022)	1/13 transplanted. No deaths	No data	>5/13 children HAdV-positive by PCR (2 on throat swab, 2 in blood and 1 in stool)
			>5/13 cases had a recent positive SARS-CoV-2 test
270 children aged 10 and under; United Kingdom Health Security Agency (UKHSA Investigation into acute hepatitis, 2022)	15/270 transplanted. No deaths	> Analysis of 6 explanted livers and 8 biopsies: Severity ranging from mild hepatocellular injury to massive hepatic necrosis, with non-specific inflammatory response	> Detection rate for adenoviral DNA: 170/258 (65.9%)
		> No evidence of adenovirus on immunohistochemistry	>4.4% of cases were positive for SARS-CoV2 in 2 weeks prior to hospital admission
		> One case underwent adenovirus PCR of liver tissue: negative	
65 children with severe hepatitis (n = 59) or PALF (N = 33) in European countries and Israel; de Kleine et al. (2022)	4 transplanted 4 deaths	No data	> Adenovirus (n = 4), SARS-CoV-2 (n = 2), SARS-CoV-2 (n = 4), rotavirus (n = 1), influenza virus type A (n = 1), EBV (n = 2), enterovirus (n = 2), rotavirus (n = 2)
5 patients with indeterminate paediatric acute liver failure (PALF)(Netherlands) Lexmond et al. (2022)	4 transplanted patients	Pt 1: massive hepatic necrosis with periportal steatotic changes; no signs of inflammatory activity	Pt 1: HAdV DNA in stool and plasma
		Pt 2: lobular disarray and spotty necrosis of remaining hepatocytes, moderate lymphocytic infiltrate	Pt 2: Plasma: HAdV DNA positive anti-SARS-CoV-2 IgG (past infection)
		Pt 3: signs of hepatitis, predominantly portal, with extensive hepatocyte loss	Pt 3: anti-SARS-CoV-2 IgG (past infection)
		Pt 5: portal and lobular inflammatory infiltrate with condensation and collapse of reticulin, some lobular disarray	Pt 5: Stool: HAdV DNA positive -recovered from SARS-CoV-2 infection 10 weeks before presentation
28 children in United Kingdom Morfopoulou et al. (2022)	5 transplanted patients	- no data	- AAV2 detected in explanted livers
			- AAV2 detected in whole blood of 10/11 non-transplanted cases
			- low levels of HAdV and HHV-6B in 5 explanted livers and blood from 15/17 and 6/9 respectively, of the 23 non-transplant cases tested

### 5.1 Adenovirus infection

**FIGURE 5:**
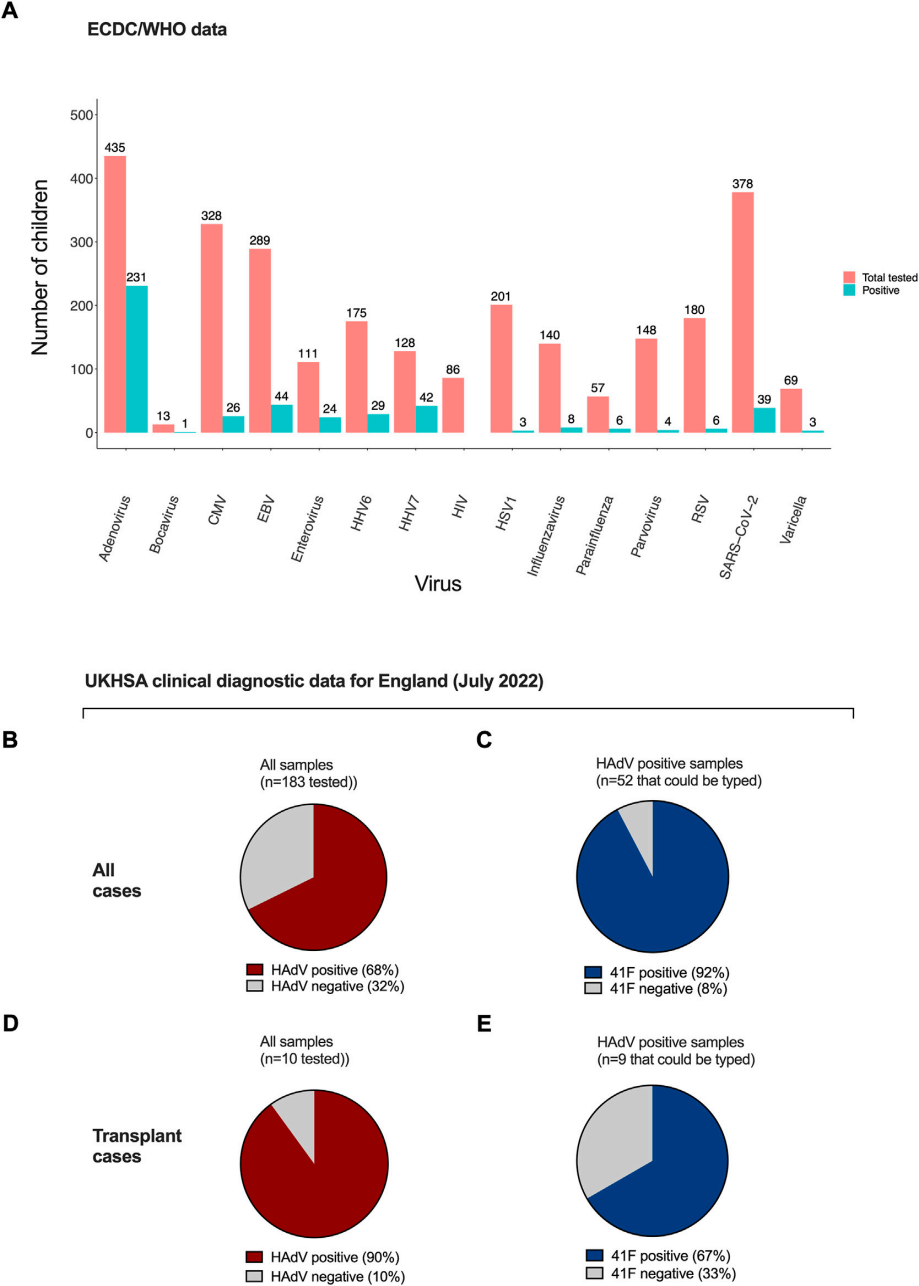
Rate of positivity for viruses among tested samples by ECDC and in UKHSA. **(A)** ECDC/WHO data for 513 samples indicating number tested and number positive (ECDC, 2022d). Panels B, C, D, E show UKHSA data reported in Technical Briefing 4 (UKHSA Investigation into acute hepatitis, 2022). Proportion of samples testing positive for HAdV in all AS-Hep-UA cases **(B)** and children undergoing transplant **(C)**. Proportion of HAdV typing as 41F in all AS-Hep-UA cases **(D)** and explant tissue **(E)**. HAdV - human adenovirus. HHV-7–human herpes virus 7, HHV-6c–human herpes virus 6, EBV—Epstein-Barr virus, CMV–cytomegalovirus, VZV—varicella-zoster virus, HSV-1–herpes simplex virus 1, RSV–respiratory syncytial virus, SARS-CoV-2–severe acute respiratory syndrome coronavirus 2.

From a total of 513 cases of AS-Hep-UA reported by 25 August 2022 (ECDC, 2022d), 404 had been tested for human adenovirus (HAdV), among which 218 (54%) tested positive. This is the highest positivity rate among all pathogens that were initially investigated as possible aetiological agents (Figure 5A) and is present in the majority of clinical samples (Figures 5B,C). Where HAdV subtyping has been carried out, subtype 41F predominates, both in nine children with AS-Hep-UA in Alabama (Baker et al., 2022; Gutierrez Sanchez et al., 2022), and in the United Kingdom, where 92% of typed HAdV infection in blood was 41F, and 67% from a small number of liver tissue samples (Figures 5D,E). HAdV 41F is typically associated with self-limiting gastroenteritis in children, and has not previously been associated with severe hepatitis. Genomic investigation has not identified any evidence of divergence from non-outbreak strains or recombination that could potentially underpin the new disease phenotype (Morfopoulou et al., 2022).

When testing for HAdV, the positivity rate was highest in NAAT in specimens of whole blood (49.3%, 140/284), followed by stool (21.8%, 55/252), respiratory samples (20.4%, 45/221), and serum (16.9%, 10/59) (ECDC, 2022d). The fact that plasma specimens have a lower positivity rate for HAdV compared to contemporaneous whole blood samples from the same patient (Baker et al., 2022; ECDC, 2022d) is important because if plasma is routinely used for testing, this may lead to underdiagnosis. Furthermore, most diagnostic PCR assays for adenovirus are not optimised for detection of group F viruses, especially in blood or plasma, so it is also conceivable that negative results reflect sub-optimal assays rather than true negativity.

HAdVs are plausible agents of AS-Hep-UA, being a recognised cause of histologically confirmed acute hepatitis. However, cases of severe HAdV-mediated liver disease in previously healthy children is unusual, as the previously described syndrome is described only in those with significant underlying immunocompromise (Hierholzer, 1992), and Ad 41F is not a previously recognised as a cause of hepatitis in children. Potentially fatal HAdV-induced fulminant liver failure has been reported in patients undergoing solid organ and haematological transplant (Norris et al., 1989; Johnson et al., 1990; Bertheau et al., 1996; Chakrabarti et al., 1999; Somervaille et al., 1999; Mihaylov et al., 2022), other chemotherapy regimens (Kaur et al., 2002), and graft-versus-host disease, and complications are highest in those with symptomatic invasive infection, involvement of more than two sites, and HAdV viraemia (Williams et al., 2009). A literature review identified a total of 89 cases of histologically confirmed HAdV hepatitis, (Ronan et al., 2014), again largely confined to immunocompromised children or neonates; serotypes 2 and 5 were the most common. In contrast to the current AS-Hep-UA outbreak, histology results in these immunocompromised children were positive for hepatic necrosis and viral inclusions (Ronan et al., 2014; Schaberg et al., 2017).

There is a knowledge-gap regarding the incidence of acute hepatitis during HAdV infection in immunocompetent hosts, particularly children, in which hepatitis is not a recognised manifestation of HAdV infection (Lynch and Kajon, 2021) [extremely rare, isolated cases have been described (Munoz et al., 1998)]. Fortunately, the high fatality rates described for disseminated adenoviral disease in high risk patients (Ronan et al., 2014; Schaberg et al., 2017) have not been seen so far in AS-Hep-UA (ECDC, 2022d).

HAdV infection as an aetiological agent in AS-Hep-UA could be confounded by increased circulation of HAdV in settings where increased numbers of AS-Hep-UA were seen (Hakim, 2022). In the United Kingdom, an increase in HAdV has been reported in faecal samples, particularly in children aged 1–4 years (GOV.UK, 2022). Appropriate case-control studies will be needed to resolve this issue. Susceptibility may be higher due to the periods of COVID-19 lockdown–particularly for young children who were not exposed to normal patterns of endemic infection (Lumley et al., 2022). However, a United States investigation found no evidence of HAdV infections in children above the expected baseline coinciding with the time period of the outbreak (Kambhampati et al., 2022).

Although the involvement of HAdV is supported by epidemiological and laboratory data, it is notable that histology from cases of AS-Hep-UA positive for HAdV did not identify the same patterns of virally-induced liver damage as those previously reported for HAdV hepatitis (Ronan et al., 2014; Baker et al., 2022), and no viral inclusions, proteins or particles have been identified by immunohistochemical staining and electron microscopy (Baker et al., 2022). Together, these data suggest that HAdV is not the sole driver of pathology in AS-Hep-UA, but is a plausible co-contributor to liver injury, together with one or more other agents, and potentially in the setting of a specific host immunological susceptibility profile, based on host genetics, or pre-existing immune activation arising from some other cause, for example triggered by a SARS-CoV-2 superantigen (Brodin and Arditi, 2022).

### 5.2 Adeno-associated viruses

**FIGURE 6:**
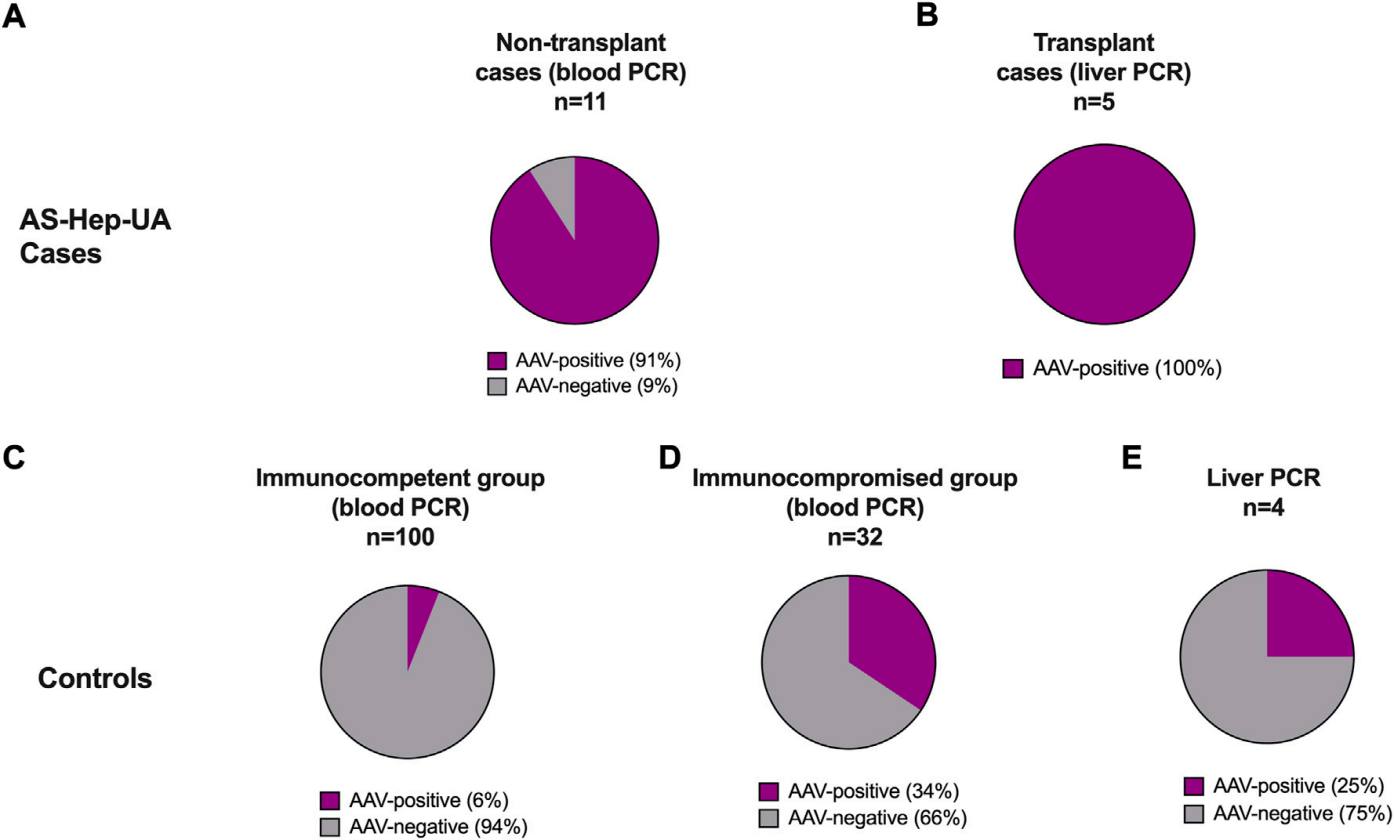
Rate of positivity for AAV among samples tested in the United Kingdom to represent AS-Hep-UA cases and a set of paediatric control samples. In each case, purple represents samples testing AAV-positive by PCR, with grey showing AAV-negative cases, demonstrating a significant excess of AAV-positives in AS-Hep-UA cases **(A,B)** compared to samples collected from children in control groups **(C–E)**. Data are collated from the report by Morfopoulou et al. (2022).

The application of metagenomic approaches and real-time polymerase chain reaction (PCR) identified AAV-2 in a high proportion of samples from children with AS-Hep-UA, including nine Scottish cases examined and 15 of 16 other cases from across the United Kingdom (Ho et al., 2022; Morfopoulou et al., 2022) (Figures 6A,B). In contrast, the majority of control samples (taken from healthy children, cases of paediatric HAdV without liver involvement, and children with hepatitis of other causes), tested negative for AAVs (Miyazawa, 2022; Morfopoulou et al., 2022) (Figures 6C–E).

Adeno-associated viruses (AAV) are Dependoparvoviruses, which are endemic in human populations but not routinely incorporated in clinical diagnostic screens for infection. AAV-2 is the most common serotype. They are defective single stranded DNA viruses that can only replicate in the presence of a ‘helper’ virus, which include Adenoviruses or human herpesvirus 6 (HHV-6). They are recognised as a rare trigger for hepatocellular carcinoma as a result of causing insertions in the human genome (La Bella et al., 2020).

Insights into pathology associated with AAVs have also developed as a consequence of these agents being used as a vector for gene therapies. Hepatotoxicity has been described (including fatal outcomes) when administered at high doses, with a hypothesis that liver injury is immunologically driven, potentially by both T cell and innate responses to the AAV capsid (Flotte, 2020; Morales et al., 2020). AAV8 has high liver tropism, but patients in whom this vector has been used are also at risk of pre-existing underlying chronic liver disease which may enhance vulnerability to hepatotoxicity (Morales et al., 2020). Dose-related hepatotoxicity may also occur as part of a multi-system syndrome, with thrombocytopaenia, nephrotoxocity and central nervous system effects also described in both human and animal studies (Hinderer et al., 2018; Flotte, 2020).

### 5.3 SARS-CoV-2

As the AS-Hep-UA outbreak has developed during the ongoing COVID-19 pandemic, a possible role for SARS-CoV-2 infection has been investigated, both considering its potential for directly causing liver injury, and *via* indirect influences (such as responses to drugs/vaccines, or changes in population immunity related to periods of lockdown). Liver dysfunction is clearly recognised in association with primary SARS-CoV-2 infection, which is mediated through multiple routes (Zhang et al., 2020), which include:

1) Direct cytotoxicity from SARS-CoV-2 replication in the liver, with evidence of SARS-CoV-2 in liver tissue samples (Yang et al., 2005) (Sonzogni et al., 2020);
2) Immune-mediated liver injury brought on by a severe inflammatory response or systemic inflammatory response syndrome (SIRS) with a ‘cytokine storm syndrome’ (Feng et al., 2020; Mehta et al., 2020);
3) Hypoxic liver damage secondary to respiratory failure or hepatic congestion from right-sided heart failure (Saeed et al., 2022);
4) Vascular damage associated with coagulopathy, endothelial inflammation, and a pro-thrombotic state (D’Ardes et al., 2022), and direct vascular involvement, with virions identified in vascular lumens and venous endothelial cells of the portal circulation (Sonzogni et al., 2020; Wang et al., 2020);
5) Upregulation of cell-surface receptors that are required for viral entry (angiotensin-converting enzyme (ACE2) and transmembrane serine protease 2 (TMPRSS2) (Paizis et al., 2005; Stertz et al., 2007; Hoffmann et al., 2020; Wang et al., 2020);
6) Bile duct injury and hepatocellular cholestasis (Geier et al., 2006) associated with hypoxia, SIRS, and viral infection of cholangiocytes;
7) Modifications to gut microbiota and damage to the gut vascular barrier (Prasad et al., 2022);
8) Drug-induced liver injury (Zhang et al., 2020).

A large study of >5700 hospitalised adults and children with SARS-CoV-2 infection reported that AST and ALT were both commonly elevated (58% and 39% of cases, respectively) (Richardson et al., 2020). However, liver enzymes would not routinely be measured in the majority of uncomplicated COVID-19 cases. When hepatitis does occur, it is typically moderate, transient, and recovers without specific treatment (Zhang et al., 2020). However, severe liver damage has been reported (Chen et al., 2020), and elevated ALT, thrombocytopaenia and hypoalbuminaemia have been associated with poor outcomes (Zhou et al., 2020; Wang et al., 2021). However, cases of AS-Hep-UA did not emerge until well beyond the peak of the pandemic, there was no evidence of direct liver infection, and most documented liver derangement in COVID-19 cases arose in adults, suggesting that SARS-CoV-2 is not the primary driver of pathology. High rates of SARS-CoV-2 exposure (based on seropositivity) have been documented in children presenting with AS-Hep-UA (Ho et al., 2022), but this is in the setting of high rates of overall community positivity, such that there was no significant difference in seroprevalence between cases and controls (UKHSA Investigation into acute hepatitis, 2022).

With the emergence of AS-Hep-UA in children, excluding any relationship with the SARS-CoV-2 vaccination became an urgent priority. However, most AS-Hep-UA cases have not received COVID-19 vaccine (due to age <5), ruling out any consistent link between cases and vaccination. As of July 2022, among 296 suspected cases from 42 states of United States, only 4.1% of the cases had received a COVID-19 vaccine (Cates et al., 2022). Likewise, European data published in August 2022 included vaccination status for 138 children, of whom 81% had not received a COVID-19 vaccine (ECDC, 2022e). COVID-19 vaccines are thus not considered possible triggers for AS-Hep-UA in children.

### 5.4 Other viral agents

In addition to HAdV and AAV, other viruses have also been identified from routine clinical analysis of laboratory samples of children with AS-Hep-UA, most commonly human herpes viruses and enteroviruses (Figure 5A). HHV-6 and -7 are notable in European and United Kingdom data, with HHV-6B detected in 5/5 livers and 6/9 blood samples in the United Kingdom investigation, but typically with high cycle threshold (CT) values (indicating low copy numbers of the virus) (Morfopoulou et al., 2022).

These data are difficult to interpret, as these viruses are ubiquitous in most populations, with primary infections commonly arising in children and adolescents, although the age of acquisition varies between settings. Latency of herpes viruses means that reactivation can occur, and may be triggered by other illnesses, while HHV-6 can also be chromosomally integrated; thus these viruses may simply be bystanders. Primary infection with HHV-6 and 7 are usually associated with mild self-limiting illness in otherwise healthy children, while acute liver failure is documented but rare (Hashida et al., 1995; Somasekar et al., 2017). Some reports of HHV-6 linked cases with fulminant hepatitis have been reported (Charnot-Katsikas et al., 2016).

Likewise, Epstein-Barr virus (EBV) and cytomegalovirus (CMV) can cause direct virus-mediated hepatocyte injury or indirect liver immunopathology (Leonardsson et al., 2017; Dunmire et al., 2018; Kwong et al., 2019), and severe or fulminant hepatitis is recognised (Kofteridis et al., 2011; Leonardsson et al., 2017; Dunmire et al., 2018) but hepatic involvement in immunocompetent hosts is usually self-limiting. Herpes simplex virus (HSV) and varicella-zoster virus (VZV) can be complicated by dissemination with acute hepatitis and liver failure (Norvell et al., 2007; Blumental and Lepage, 2019; Little et al., 2019; Spahn et al., 2022), but the low prevalence of these viruses in AS-Hep-UA cases does not favour causality and there is a high chance that these viruses are bystanders. Enteroviruses are a recognised cause of hepatitis (ECDC, 2022e), sometimes with a severe rebound after initial improvement, in immunocompetent children and in patients receiving anti-CD20 agents and in neonates, who have an immature B-cell repertoire (Abzug, 2001; Tebruegge and Curtis, 2009; Morgan et al., 2015; Antona et al., 2016; Bajema et al., 2017; Nicolini et al., 2019). Human parainfluenza viruses (HPIV), bocavirus, influenza virus and parvovirus have been detected in a small proportion of AS-Hep-UA cases (Figure 5A), but these are deemed more likely to be bystanders than aetiological agents.

## 6 Immunological aetiology of AS-Hep-UA

### 6.1 Evidence for immune aetiology

Limited histopathological data from liver biopsies failed to identify specific evidence of viral infection, suggesting an indirect mechanism for the hepatic injury, possibly related to immune dysregulation. Multiple immunological drivers can contribute to a severe outcome during acute hepatitis in children, including host genetic susceptibility, a hyperinflammatory reaction to prior, acute or chronic infection, an autoimmune response triggered by a viral or an environmental factor, or a superantigen reaction prompting T cell activation. SARS-CoV-2 spike protein has been proposed as a superantigen motif (analogous to Staphylococcal enterotoxin B) which could trigger non-specific T-cell activation, particularly if long-term reservoirs of SARS-CoV-2 persist (Brodin and Arditi, 2022).

An impaired cellular and humoral immune response, as well as an antigen driven abnormal T cell immune activation have previously been reported in cases of paediatric acute liver failure (PALF) of unknown aetiology (Squires et al., 2022). Dense infiltrates of clonal, activated CD8^+^ T cells are frequently found in liver biopsies from these cases and have been proposed as a biomarker for this liver disease phenotype (Chapin et al., 2020). In addition, dynamic networks of inflammatory mediators have been described in children with acute liver failure, with distinct Th1 response patterns associated with disease evolution (Azhar et al., 2013; Chapin et al., 2020).

In AS-Hep-UA cases, simultaneous or consecutive viral infections (e.g., AAV with either Adenovirus or HHV-6) might have triggered severe hyperinflammatory responses in the liver. Such mechanisms have been suggested in the multisystem inflammatory syndrome in children (MIS-C) that can occasionally complicate SARS CoV-2 infection in children and is described during haemophagocytic lymphohistiocytosis (HLH), an excessive immune activation syndrome. In these cases, liver injuries (hepatitis or hepatomegaly associated with fever) have been described, in association with hyperactivation and proliferation of T cells, macrophage activation and overproduction of inflammatory cytokines (Filipovich and Chandrakasan, 2015; Hoste et al., 2022).

The majority of experimental data on the role of cytokines in the pathogenesis of adenovirus infections comes from mouse models. Elimination of adenoviral vectors *in vivo* is mediated by CD8^+^ T-cells, NK-cells and CD4^+^ T-cells. A mouse model of innate immune response to replication-defective adenovirus emphasized a key role of NK-cells, neutrophils and Kupffer cells in the development of acute liver toxicity (Ajuebor et al., 2008). Activated hepatic γδ T-cells that interact with hepatocytes and induce local production of CXCL9, may act as a chemotactic signal to induce accumulation of CXCR3-positive γδ T-cells into the liver, mediating acute liver damage without vector clearance. In addition, adenoviral infection increases the severity of liver injury following exposure to staphylococcal enterotoxin B *in vivo* and *in vitro*, mediated by IFN-γ (Yarovinsky et al., 2005).

AS-Hep-UA appears to be driven by indirect immune-mediated mechanisms, as there is no evidence of viral infection of the liver. Due to the very limited data on the expression of biological response mediators in children with AS-Hep-UA, measurement of cytokine and chemokine concentrations in blood and tissue by multiplex-based platforms (such as bead-based flow cytometry or Luminex-based technologies) could help identify signature molecular patterns that might provide a better insight into the exact immunopathogenic mechanisms of this disease.

### 6.2 Host genetic susceptibility

Specific host alleles may favour the development of a disproportionate inflammatory response. A specific HLA class II allele, DRB1*04:01 has been identified in the majority of cases of acute hepatitis in children in Scotland and England who had signs of coinfection with HAdV and AAV2 in the liver, suggesting an immune mediated aetiology in a particular genetic background (Ho et al., 2022; Morfopoulou et al., 2022). This allele has a reported prevalence of 10–13% in England and Scotland (AFND, 2022). Alleles at the DRB1 locus have previously been associated with a variety of autoimmune diseases, including hepatic autoimmunity (Terziroli Beretta-Piccoli et al., 2022), although specific autoantibodies in children in this outbreak have been negative (Ho et al., 2022).

## 7 Other aetiological hypotheses

### 7.1 Food/water borne aetiology

Food or water-borne transmission is a potential route of spread for hepatitis outbreaks. Although foodborne outbreaks would be expected to affect all ages, young children may be disproportionately affected—for example, a recent *Salmonella typhimurium* outbreak mainly affected children aged <10 years (Larkin et al., 2022). The possibility of a non-infectious foodborne agent has been considered (Salmon and Palmer, 2022), e.g., mycotoxins such as amatoxin and aflatoxin. The pathology seen in AS-Hep-UA patients is compatible with mycotoxins, and some mycotoxins were detected in clinical samples but such toxins are also detected in controls, making a causative association unlikely. Furthermore, the worldwide distribution of AS-Hep-UA cases, with no clear geographic links between cases makes it less likely that a specific toxin or contamination of food or water is involved.

### 7.2 Drug-induced liver injury

DILI has been explored as a possible cause of AS-Hep-UA in children. DILI can be related to prescribed medications (Molleston et al., 2011; Lee, 2013), over-the-counter drugs, or herbal/traditional remedies [including products containing pyrrolizidine alkaloids, germander, ma Huang, chaparral, black cohosh root, pennyroyal, and kava (Stickel et al., 2005; Stirnimann et al., 2010)], with effects arising from the parent drug or its metabolites, together with a host immune response.

Diagnosis of DILI can be challenging, as onset of liver injury can be delayed (Chalasani et al., 2021), and >1000 medications and herbal products have been implicated to date (EASL Clinical Practice Guidelines: Drug-induced liver injury, 2019). In the absence of diagnostic tests and biomarkers, DILI requires a high index of suspicion and is mostly a diagnosis of exclusion based on detailed history and exclusion of other causes (Lucena et al., 2001; Navarro and Senior, 2006; Yuan and Kaplowitz, 2013; Kleiner, 2014; Chalasani et al., 2015).

Although epidemiological investigations are still underway, in September 2022 the United States CDC states no associations have been found between cases of AS-Hep-UA and animal contact, food, medication, toxins, or other exposures (CDC, 2022).

## 8 Discussion

The working hypothesis for the aetiology of this outbreak of acute severe hepatitis in children (Figure 1B) is that a combination of AAV-2 infection in the presence of a helper virus (probably HAdV-41F or HHV-6) triggers an aberrant immune response in children who are made susceptible by an HLA allelic variant. The outbreak may also relate to vulnerability in children due to changes in population exposure and immunity as a result of periods of pandemic lockdown in 2020 and 2021 (Lumley et al., 2022), which may have left young children uniquely susceptible. However, existing data do not categorically confirm causality, and there remain some significant challenges and unanswered questions, which we review further in this section.

The lack of data and incomplete understanding of the aetiology and pathogenesis of this new disease may deprive affected children of the use of possibly effective targeted therapies to improve outcomes and potentially even prevent the need for liver transplantation, highlighting the need for ongoing collaboration, investigation and vigilance.

### 8.1 Challenges for epidemiological data collection

Thorough global investigation is hampered by the heterogeneity of clinical and laboratory data, according to the resources, capacity, infrastructure and data collection systems available in different settings. The small case numbers reported from some regions genuinely reflect an absence of any outbreak, but it is also possible that cases have been missed due to limited access to clinical care and diagnostics, and inadequate surveillance systems. Detailed investigations often cause delays, meaning there can be a lag between case presentation and data reporting (GOV.UK, 2022). As this wave of paediatric hepatitis cases wanes, attention of public health and research communities has diverted to competing public health priorities including investment in recovery from the impact of the ongoing COVID-19 pandemic, and focus on the subsequent rise in monkeypox cases as a Public Health Emergency of International Concern (Benites-Zapata et al., 2022).

Conversely, there is also a risk of over-reporting, as enhanced scrutiny has been applied to all cases of acute hepatitis, even though absolute case numbers have not exceeded the background incidence of paediatric hepatitis in many settings. To date, the United States CDC has ruled out an outbreak (Kambhampati et al., 2022) and there is no clear evidence of cases exceeding the background rate of acute hepatitis outside the United Kingdom and selected European countries.

Differing epidemiology of childhood infection between settings may explain genuine differences in susceptibility, and can also influence immune ontogeny in early life (e.g., almost 100% of African children acquire CMV infection within the first year of life; exposure to malaria may alter immune responses to other infections). This influence operates alongside population genetic structures in which the prevalence of alleles associated with either risk or protection are different. The extent to which population lockdown has been a significant influence in modulating immunity in children remains unknown, but we should remain vigilant for the consequences of delays in exposure to common childhood infections, as well as the potential disruption to routine vaccination schedules.

### 8.2 Laboratory challenges

There are challenges in identifying potential pathogens that are not detected by routine laboratory diagnostic tests (in this case, exemplified by AAV). Generating metagenomic data is typically expensive, both in hardware costs to set up, and in maintenance, consumables, and the development of appropriate analysis pipelines. However, new approaches may reduce costs, and the COVID-19 pandemic has illustrated the potential for rapid scale up of infrastructure–with Africa leading on international genomics surveillance, and analysis code being widely shared. Nanopore sequencing technology offers the potential for a point of care approach to sequencing, reducing the infrastructure demands of generating metagenomic data.

Interpretation of metagenomic data is challenging, as the complexity of the human microbiome, together with the sensitivity of the approach, can make it difficult to distinguish between pathogens, bystanders, commensal flora, and contaminants. For this reason, shared approaches to analysis and interpretation of metagenomic data sets is important to bring consistency between settings (Magiorkinis et al., 2019). In this hepatitis outbreak, interpretation is further compounded by the potential role of common viruses presenting with uncommon manifestations, the role of viruses that are long term passengers and that can reactivate in the setting of other acute illness, and by the likely role of co-infection such that causality is driven by (potentially varied) combinations of agents.

The sensitivity and specificity of laboratory diagnosis is dependent on sample type tested, the timing of sample collection, and preservation methods. For example, for systemic infections, viral detection is most sensitive if whole blood is tested. In this case, clear understanding of the disease pathogenesis is dependent on access to liver tissue, which requires children to be managed in a setting in which liver biopsy can be safely undertaken.

### 8.3 Clinical interpretation and intervention

Although there is a signal for the role of HAdV, this has not been consistently identified in all cases in this outbreak, and there are no data to support direct replication in liver tissue. Severe HAdV disease in immunocompetent hosts beyond the neonatal period has not been previously recognised. Because most HAdV infections are mild and self-limiting, there are few pre-existing data on important biological features, for example the proportion of infected children who are viraemic, the proportion who develop liver dysfunction (either clinical or subclinical), and any specific risk factors for complications. All of these data gaps are exaggerated when considering the specific issue of enteric HAdV infection. There are limited data on the role of AAV as pathogens or co-pathogens, although use of AAV as vectors has provided evidence of triggering of inflammatory pathways in the liver.

An understanding of pathophysiology is integral to informing interventions. One consideration is the extent to which antiviral treatment may be of benefit, particularly uncertain if there is no evidence of viraemia or infection of liver tissue, which suggests the primary infection may have already abated before the development of immunopathology. An alternative treatment consideration is immunomodulatory therapy to curtail immune activation and reduce the associated liver injury. Robust clinical trials would require the enrollment of sufficient case numbers to power different endpoint analyses, which has not been possible within this outbreak.

At a population level, ongoing surveillance will be required to determine whether further sporadic cases arise, or whether there is resurgence of an outbreak. WHO advises basic hygiene methods for prevention, such as handwashing, assurance of safe drinking water, and general respiratory precautions (WHO, 2022d), but these are non-specific and it is not known to what extent these general measures will have specific impact on risk reduction, particularly in children < age five in whom enforcement of behavioural strategies can be challenging.

Case-control studies have been conducted based on the comparison of children with AS-Hep-UA versus children presenting with confirmed HAdV infection (without hepatitis) or hepatitis (of other known causes), but there are difficulties in assimilating comparable age-matched control groups [controls are typically older than cases (Morfopoulou et al., 2022)], and access to liver tissue may be limited. As rapid and/or molecular diagnosis tools are becoming more widely available in clinical practice, more data are expected to be gathered, gradually distinguishing the characteristics of different biological agents that may cause one common clinical syndrome.

Due to the diversity of the host genetic patterns and various aetiological agents detected in individual patients, the analysis of biological response modifiers might benefit from more advanced bioinformatics approaches, including exploratory modelling analyses of chemokine and cytokine panels associated with innate and specific immune responses (including Th1, Th2, Th9, Th17, Th22 cytokines). This approach might help identify clustering of immune mediators in particular groups of patients based on the severity of clinical presentation or patterns of verified aetiology. Furthermore, if the primary underlying mechanism for liver injury is immune mediated, immunomodulatory therapy may be a valuable therapeutic tool, and identification of specific biomarkers may contribute to the early identification of patients who will benefit most from such intervention.

### 8.4 Lessons learned for pandemic preparedness

This hepatitis outbreak highlights again the lessons being learned from the COVID-19 pandemic, with an urgent need for unified rapid and responsive surveillance systems supported by sustainable resources, and the need to improve equity with enhanced capacity building in resource-limited settings. We should consider whether early warning systems could be improved to alert the international community to future outbreaks, through algorithms that can identify changing patterns of disease presentation, either through coding or through laboratory tests.

### 8.5 Summary/conclusions

Rapid investigations of an outbreak of severe hepatitis in children have led to a working paradigm to explain likely causality, pointing to one or more viral infections that may trigger severe hyperinflammatory responses in the liver, most probably in previously immunologically naïve children who have a particular genetic background. However, there are unanswered questions about epidemiology, pathophysiology and optimum approaches to laboratory, clinical and public health interventions for this outbreak, and the experience highlights an ongoing need for global collaboration to enhance outbreak preparedness.

## Glossary

AAV: Adeno-associated virus
ACE: Angiotensin converting enzyme
ALT: Alanine transferase
Anti-HBc IgM: Anti-Hepatitis B core immunoglobulin M
AS-Hep-UA: Acute severe hepatitis of unknown aetiology
AST: Aspartate transferase
**COVID**-19: Coronavirus disease 2019
CMV: Cytomegalovirus
CT: Cycle threshold
DILI: Drug-induced liver injury
EBV: Epstein-Barr virus
ECDC: The European Centre for Disease Prevention and Control
Epi-linked: Epidemiologicall linked
ESCMID: The European Society for Clinical Microbiology and Infectious Diseases
ESGVH: ESCMID Study Group for Viral Hepatitis
EU/EEA: The European Union/European Economic Area
HAdV: Human adenovirus
HAV: Hepatitis A virus
HBsAg: Hepatitis B surface antigen
HBV: Hepatitis B virus
HCV: Hepatitis C virus
HDV: Hepatitis D virus
HEV: Hepatitis E virus
**HHV** Human herpesvirus
HLA: Human leukocyte antigen
HLH: Haemophagocytic lymphohistiocytosis
HPIV: Human parainfluenza virus
HSV: Herpes simplex virus
IgM: Immunoglobulin M
IQR: Interquartile range
MIS-C: Multisystem inflammatory syndrome in children
NAAT: Nucleic acid amplification test
NK-cells: Natural killer cells
PALF: Paediatric acute liver failure
PCR: Polymerase chain reaction
**SARS-CoV-2**: Severe acute respiratory syndrome coronavirus 2
SIRS: Systemic inflammatory response syndrome
TESSy: The European Surveillance System
TMPRSS2: Transmembrane serine protease 2
UK: The United Kingdom
UKHSA: The United Kingdom Health Security Agency
USA: The United States of America
US CDC: The United States´Centers for Disease Control and Prevention
VZV: Varicella-Zoster virus
WHO: The World Health Organization
